# Operative Outcomes and Training Equity in Appendicectomy: Are Core Surgical Trainees Getting a Fair Chance?

**DOI:** 10.7759/cureus.98210

**Published:** 2025-12-01

**Authors:** Ananya Nayak, Obed Amoako-Adjei, Rabia Arshad, William Down, Alexia Defer, Bishow Karki, Alexander Wilkins

**Affiliations:** 1 General Surgery, Mid Yorkshire Teaching NHS Trust, Wakefield, GBR; 2 General Surgery/Trauma and Orthopaedics, Mid Yorkshire Teaching NHS Trust, Wakefield, GBR; 3 General Surgery, The Suture Centre, Hull Surgical Skills Training Centre, Hull University Teaching Hospital NHS Trust, Hull, GBR

**Keywords:** appendicectomy, core surgical training, emergency general surgery, training, united kingdom

## Abstract

Introduction: Appendicectomy is an important index procedure for core surgical trainees (CSTs). However, there are growing concerns that reduced operative exposure due to service pressures and working time restrictions may be limiting CST experience. This study aimed to assess the extent of CST involvement in appendicectomies at a district general hospital in the United Kingdom and to compare the safety, efficacy, and outcomes of procedures performed across different grades of surgeons.

Methods: A retrospective review was conducted of all appendicectomies performed between December 2023 and June 2024. Demographic, perioperative, and postoperative data were extracted from electronic records. Patients undergoing appendicectomy for non-appendicitis indications were excluded. Cases were categorised by the primary operator: consultant, registrar, or CST. Data were analysed using IBM SPSS Statistics for Windows, version 26 (IBM Corp., Armonk, New York, United States). Continuous and categorical variables were compared across surgeon grades using appropriate parametric or non-parametric tests. Variables with significant or clinically relevant differences were entered into multivariable logistic regression models adjusting for key confounders, with results reported as odds ratios (ORs) and 95% confidence intervals (CIs). A p-value* *<0.05 was considered statistically significant.

Results: A total of 254 appendicectomies were analysed: 46 (18.1%) consultant-led, 170 (66.9%) registrar-led, and 38 (15.0%) CST-led. Median patient age was 31 years (IQR 29), with no significant difference in gender or American Society of Anesthesiologists (ASA) physical status distribution across groups. Consultants operated on older patients with higher pre-operative C-reactive protein (CRP) levels (p < 0.001), reflecting more complex cases. Case complexity varied significantly by operator grade (p = 0.012). Operative duration, length of stay, complication rate, and readmission rate were similar across all grades (p > 0.05). In adjusted analyses, surgeon grade was not an independent predictor of complications (CST vs consultant: adjusted OR 2.85, 95%CI 0.67-12.00, p > 0.05) or readmissions (CST vs consultant: adjusted OR 1.70, 95%CI 0.34-8.64, p > 0.05).

Conclusion: CSTs performed significantly fewer appendicectomies than registrars yet achieved comparable outcomes when given the opportunity. No differences were found in operative duration, complication, or readmission rates between grades. These findings support increased supervised autonomy and equitable case allocation for CSTs to ensure adequate operative exposure and preparedness for higher surgical training. National evaluation of trainee involvement in index procedures is warranted to optimise training and workforce sustainability.

## Introduction

Acute appendicitis is the most common surgical emergency in the world. It is estimated that around 50,000 appendicectomies are performed in the United Kingdom (UK) annually [1]. The admission and subsequent operation for these patients is very often conducted by resident doctors, either supervised or unsupervised by a consultant.

Appendicectomy is traditionally regarded as a benchmark emergency general surgical procedure and an essential index case for UK core surgical trainees (CSTs), and the Joint Committee on Surgical Training (JCST) outlines expected competence in core laparoscopic emergency procedures before progression to higher surgical training [2]. Operative experience is not only relevant to technical development but also directly influences selection into higher surgical training, where logbook numbers and procedural competency are key determinants of shortlisting [3]. CSTs are expected to have performed more than 36 appendicectomies to gain maximum points in higher surgical training applications [4].

CSTs in the UK are generally in postgraduate year (PGY) 3-4, following two years as foundation doctors (PGY1-2). Registrars are in higher surgical training and typically complete at least six additional years (PGY5-10) before they can apply for a consultant role. A lack of early exposure may undermine preparedness for registrar-level on-call duties and threaten workforce sustainability in general surgery. There have also been concerns that the implementation of the European working time directive has reduced the number of operations by residents and thus affected the quality of care they would deliver [5]. Studies relating to such concerns have largely been conflicting [6-9].

This study aimed to assess the extent to which trainees of the core training grade are performing appendicectomies in a UK district general hospital and to evaluate whether their operative performance and outcome differ from those of registrars and consultants. By examining case complexity, outcomes, and training opportunities, this study seeks to contribute to ongoing discourse on whether the current CST programme remains fit for purpose.

The project was presented as an oral presentation at the 49th Association of Surgeons in Training (ASiT) Annual Conference in Belfast on March 8, 2025, where it was nominated for the ESO Education and Training Prize.

## Materials and methods

This was a retrospective study conducted at Pinderfields Hospital, Aberford Road, Wakefield, United Kingdom. This study involved a retrospective analysis of anonymized patient records. In accordance with UK Health Research Authority (HRA) guidelines, formal ethical approval and individual patient consent were waived, as this was a service audit. Institutional approval was obtained from Mid Yorkshire Teaching NHS Trust on September 26, 2025.

Study population

All emergency appendicectomies performed at our hospital for acute appendicitis between December 2023 and June 2024 were included in the study. Cases were excluded if the appendicectomy was performed as part of another surgical procedure (e.g, hysterectomy, colectomy), during a re-look operation, or as a stump appendicectomy. Our surgical department is part of a teaching hospital and has 10 CSTs, 16 registrars, and 18 consultants on the on-call roster. We perform adult appendicectomies and paediatric appendicectomies in children older than five years. 

Standard practices

In our hospital, there are no formal criteria that determine when a resident is permitted to perform an appendectomy independently. A resident may carry out the procedure without supervision if the on-call surgeon deems them competent to do so. The resident’s training level is generally not the deciding factor, although in practice, more experienced residents are typically the ones who operate unsupervised. As a teaching hospital, we aim for residents to perform surgeries whenever possible, either independently or under the supervision of a qualified surgeon.

Imaging, such as ultrasonography or computed tomography (CT), is added to the standard assessment based on clinical judgment. If these investigations are inconclusive and there remains a strong suspicion of appendicitis, a diagnostic laparoscopy is undertaken. Laparoscopic appendectomy is the intended approach unless contraindications are present. The decision to convert from a laparoscopic to an open procedure is made intraoperatively. When a resident is operating without direct supervision, this decision is usually taken after consultation with the on-call surgeon.

Data collection

All patient information is recorded electronically on Cito (Civica Group Limited, London, UK) and PPM+ (Mid Yorkshire Hospitals NHS Trust) patient electronic record systems within our trust and was reviewed retrospectively. These variables included age, sex, Society of Anesthesiologists (ASA) physical status grade, preoperative white cell count and C-reactive protein (CRP), length of stay, readmissions, and complications. Data such as the date, start time, and operative duration were extracted from the theatre software AQUA-Theatreman (Trisoft Ltd., Nottingham, UK). Operative details such as grade of surgeon (consultant, registrar, CST), intraoperative findings, conversions, and drain insertion were taken from the surgical notes.

Complications were classified using the Clavien-Dindo system [10]. Any complication occurring during follow-up, either within 30 days of the operation or later if clearly related to the appendectomy, was included. In-hospital mortality was defined as death occurring during the admission or within 30 days after discharge. The data was independently reviewed by three different reviewers to ensure accuracy and reduce bias. 

Data analysis

All data were analysed using the IBM SPSS Statistics for Windows, version 26 (Released 2018; IBM Corp., Armonk, New York, United States). Continuous variables were assessed for normality and presented as mean ± standard deviation (SD) or median with interquartile range (IQR), as appropriate. Comparisons of continuous variables between surgeon grades were performed using one-way analysis of variance (ANOVA) for normally distributed data and the Kruskal-Wallis test for non-normally distributed data. Categorical variables were compared using the Chi-square (χ²) test or Fisher’s Exact test when expected cell counts were <5. Variables showing significant or clinically relevant differences were included in multivariable logistic regression models to evaluate predictors of postoperative complications and hospital readmissions. The models adjusted for potential confounders, including patient age, ASA grade, and intraoperative pathology (case complexity). Results were expressed as odds ratios (ORs) with 95% confidence intervals (CIs), and a two-tailed p value <0.05 was considered statistically significant.

## Results

A total of 254 patients who underwent appendicectomy were included. Of these, 46 (18.1%) procedures were performed by consultants, 170 (66.9%) by registrars, and 38 (15.0%) by CSTs. There was a slightly higher proportion of male (n=140, 51.2%) compared to female (n=124, 48.8%) patients. The mean age was 34.7 ± 18.2 years, ranging from six to 87 years. Patient age and preoperative CRP differed significantly among operator grades (p = 0.021 and p < 0.001, respectively), while other baseline characteristics were comparable (Table 1).

**Table 1:** Baseline patient characteristics by surgeon grade (Total patients: 254) F= one-way ANOVA; H = Kruskal–Wallis test; χ² = Chi-square test; df = degrees of freedom, shown in parentheses, e.g., χ²(2) indicates a Chi-square test with 2 degrees of freedom; Fisher's exact test used when expected cell count is less than 5 CRP: C-reactive protein; ASA: American Society of Anesthesiologists; IQR: interquartile range; pre-op: preoperative; CST: core surgical trainee

Characteristics	Operator characteristic			Statistic	P
	Consultant	Registrar	CST		
Number of patients	46	170	38		
Age, median (IQR)	41 (19.5-63)	31 (19-45.75)	24.5 (20.25- 42)	F(2, 251) = 3.94	0.021
Sex, n (%)				χ²(2) = 1.26	0.53
Male	28 (60.9%)	85 (50.0%)	17 (44.7%)		
Female	18 (39.1%)	85 (50.0%)	21 (55.3%)		
ASA grade, n (%)				Fisher's exact	0.3
Grade I	13 (28.3%)	58 (34.1%)	15 (39.5%)		
Grade II	28 (60.9%)	105 (61.8%)	23 (60.5%)		
Grade III	5 (10.9%)	7 (4.1%)	0 (0.0%)		
Pre-op Hb (mg/dL), mean±SD	138.9±15.1	139.1±14.4	139.9±13.2	F(2, 251) =0.047	0.954
Pre-op white blood cell count (x10^3^ cells/μL), median (IQR)	12.5 (9.5-15.7)	12.8 (9.2-16.5)	10.6 (7.8-13.8)	H(2) = 1.86	0.105
Pre-op CRP (mg/L), median (IQR)	78.5 (12.8-181.5)	27 (7-70)	57 (16-121)	H(2) = 13.73	<0.001

Case complexity and operative characteristics

The distribution of intraoperative findings differed significantly between surgeon grades (p = 0.012, Fisher’s exact test), suggesting variation in case complexity across operator levels (Table 2). Of consultant-operated cases, 34.7% were appendicular masses. The mean operative time was 83 minutes for consultant-led operations, 78 minutes for registrar-led operations, and 85 minutes for CST-led operations. No significant differences were observed in duration of surgery (p = 0.094), length of stay (p = 0.061), complication rates (p = 0.23), or readmission rates (p = 0.49) among the three groups (Table 3). Surgical duration increased with case complexity, being shortest for normal appendices and longest for perforated or mass-forming cases. Drain insertion was significantly more common in inflamed and perforated cases (p < 0.001), as shown in Table 4.

**Table 2:** Association between surgeon grade and intraoperative findings CST: core surgical trainee

Case complexity	Consultant, n (%)	Registrar, n (%)	CST, n (%)	Statistic	p-value
Normal	3 (6.5)	13 (7.6)	6 (15.8)	Fisher's exact Test	0.012
Inflamed	22 (47.8)	110 (64.7)	26 (68.4)		
Mass	16 (34.7)	43 (25.3)	5 (13.1)		
Perforated	5 (10.8)	3 (1.7)	1 (2.6)		

**Table 3:** Operative characteristics by surgeon grade χ² = Chi-square test;  F = one-way ANOVA; Fisher’s Exact = Fisher’s exact test used when expected cell counts <5. Degrees of freedom (df) are indicated in parentheses — e.g., χ²(2) denotes a Chi-square test with 2 df. CST: core surgical trainee

Characteristics	Operator characteristic			Test Statistic	P
	Consultant (n=46)	Registrar (n=170)	CST (n=38)		
Duration of surgery in minutes, median (IQR)	83.5 (62.5-116.7)	80 (61-98)	81 (71-90.75)	F(2, 251) = 2.13	0.094
Length of stay (days), mean±SD	4.1±2.5	3.1±2.4	3.4±2.5	F(2, 251) = 2.82	0.061
Complications, n (%)					
Yes	5 (10.9%)	15 (8.8%)	6 (15.8%)	χ²(2) = 3.46	0.23
No	41 (89.1%)	155 (91.2%)	32 (84.2%)		
Severity of complications (Clavien Dindo Grading), n (%)					
Grade 1	2 (4.3%)	7 (4.1%)	0 (0%)	Fisher’s Exact	0.43
Grade 2	3 (6.5%)	5 (2.9%)	6 (15%)		
Grade 3	0 (0%)	2 (1.2%)	0 (0%)		
Grade 4	0 (0%)	1 (0.6%)	0 (0%)		
Readmissions, n (%)					
Yes	3 (6.5%)	9 (5.3%)	4 (10.5%)	Fisher’s Exact	0.49
No	43 (93.5%)	161 (94.7%)	34 (89.5%)		

**Table 4:** Comparison of surgical duration and drain insertion by pathology and surgeon grade H = Kruskal–Wallis test; Fisher's exact test has been used when cell count is less than 5 CST: core surgical trainee

Characteristics		Operator characteristic			Test statistic	P
		Consultants	Registrars	CST		
Normal appendix	Duration of operation (minutes), median (IQR)	71 (63-75)	72 (60-89)	79.5 (60-83.25)	H(2) = 0.16	0.921
	Drain insertion, n (%)					
	Yes	1 (33.3%)	0 (0.0%)	1 (16.7%)	Fisher's exact	0.13
	No	2 (66.7%)	14 (100.0%)	5 (83.3%)		
Inflamed appendix	Duration of operation (minutes), median (IQR)	67.5 (57-10.25)	71 (56.5-86.5)	80.5 (71.7-93.2)	H(2) = 1.62	0.445
	Drain insertion, n (%)					
	Yes	8 (36.4%)	8 (7.3%)	2 (7.7%)	Fisher's exact	<0.001
	No	14 (63.6%)	102 (92.7%)	24 (92.3%)		
Perforated appendix	Duration of operation (minutes), median (IQR)	104 (77.5-121.5)	106 (84-125.5)	90 (71-130)	H(2) = 0.36	0.834
	Drain insertion, n (%)					
	Yes	14 (87.5%)	31 (72.1%)	3 (60.0%)	Fisher's exact	0.35
	No	2 (12.5%)	12 (27.9%)	2 (40.0%)		
Appendicular mass	Duration of operation (minutes), median (IQR)	129 (95-148)	82 (77-92)	88 (only one case)	H(2) = 1.59	0.450
	Drain insertion					
	Yes	3 (60.0%)	3 (100.0%)	0 (0.0%)	Fisher's exact	0.16
	No	2 (40.0%)	0 (0.0%)	1 (100.0%)		

Complications and re-admissions

Complication rates were compared across surgeon grades using both unadjusted and multivariable logistic regression models. The “Mass” pathology subgroup was excluded from regression analyses due to low case numbers, which caused unstable model estimates. In the unadjusted analysis, there were no statistically significant differences in complication rates across surgeon grades. Compared with consultant-led procedures, registrar-led operations had lower odds of complications (OR 0.68, 95%CI 0.23-2.01, p > 0.05), while CST-led operations had higher odds (OR 1.54, 95%CI 0.43-5.50, p > 0.05; Table 5). After adjusting for age, ASA grade, and pathology, surgeon grade remained non-significant. Registrars had lower adjusted ORs (aOR) of 0.74 (95% CI 0.23-2.40), p > 0.05, and CST-led procedures had higher odds (aOR 2.85, 95%CI 0.67-12.00, p > 0.05) (Table 6). Neither age, ASA, nor pathology significantly influenced complication risk.

**Table 5:** Unadjusted logistic regression of surgeon grade and postoperative complications (excluding “Mass” pathology) Reference category = Consultant CST: core surgical trainee

Variable	Odds Ratio	95% CI	p-value
Registrar vs Consultant	0.68	0.23 – 2.01	> 0.05
CST vs Consultant	1.54	0.43 – 5.50	> 0.05

**Table 6:** Multivariable logistic regression including surgeon grade, age, ASA, and pathology (excluding “Mass” pathology) Reference categories = Consultant (Grade) and Normal (Pathology) CST: core surgical trainee; ASA: American Society of Anesthesiologists

Variable	Adjusted OR	95% CI	p-value
Registrar vs Consultant	0.74	0.23 – 2.40	> 0.05
CST vs Consultant	2.85	0.67 – 12.00	> 0.05
Age (per year)	1.01	0.98 – 1.05	> 0.05
ASA (per point)	0.88	0.30 – 2.59	> 0.05
Pathology			
Inflamed vs Normal	0.51	0.09 – 2.77	> 0.05
Perforated vs Normal	3.32	0.60 – 18.42	> 0.05

Readmission rates were similarly low and did not differ significantly between grades (p=0.49, Fisher's exact test). The “Mass” pathology subgroup was again excluded from regression models due to small numbers. Unadjusted analysis showed no significant association between surgeon grade and readmission: registrars had lower odds (OR 0.80, 95%CI 0.21-3.09, p > 0.05) and CSTs had higher odds (OR 1.69, 95%CI 0.35-8.05, p > 0.05) compared with consultants. After adjusting for age, ASA grade, and pathology, no independent predictors of readmission were identified (Table 7). Registrars had a lower aOR of 0.65 (95%CI 0.16-2.62), p > 0.05, and CSTs had a higher aOR of 1.70 (95%CI 0.34-8.64), p > 0.05, compared with consultants. Postoperative complications and readmission rates were similar across surgeon grades. After controlling for patient ASA status, age, and intraoperative pathology, surgeon grade was not an independent predictor of complications or re-admissions.

**Table 7:** Multivariable logistic regression for predictors of readmission (excluding “Mass” pathology) Reference categories = Consultant (Grade) and Normal (Pathology) CST: core surgical trainee; ASA: American Society of Anesthesiologists

Variable	Adjusted OR	95% CI	p-value
Registrar vs Consultant	0.65	0.16 – 2.62	> 0.05
CST vs Consultant	1.70	0.34 – 8.64	> 0.05
Age (per year)	1.00	0.96 – 1.04	> 0.05
ASA (per grade)	0.76	0.22 – 2.58	> 0.05
Pathology			
Inflamed vs Normal	1.60	0.18 – 13.77	> 0.05
Perforated vs Normal	2.58	0.26 – 25.86	> 0.05

## Discussion

We assessed the duration, patient characteristics, and complications after appendicectomy by CSTs, registrars, and consultants in this study. We found that the difference in duration of surgery between the various grades was not statistically significant. We also found no evidence to suggest that patients operated on by CSTs or registrars have a greater risk of complications. Our findings are in line with several other studies, which have shown that appendicectomies performed by residents are safe [11-17]. 

A significant number of normal and inflamed appendicectomies were performed by consultants. This is likely due to service pressures where core trainees and registrars are often expected to perform multiple other tasks in addition to operating. Appendicectomy is one of the first operations that is performed by residents with and without supervision [18]. Our findings suggest that this needs to remain the case and will ensure adequate training. Evidence also suggests that adverse outcomes, such as infectious complications and re-admission rates, are lower at teaching institutions [14]. 

Over a third of core trainees do not meet their operative Annual Review of Competency Progression (ARCP) requirements annually [19]. Appendicectomy, historically the backbone of emergency surgical training, is increasingly allocated to registrars for efficiency and service continuity. Reduced exposure risks undermining technical competence and confidence during progression to ST3. This threatens equitable access to higher surgical training and compromises preparedness for emergency general surgery registrar roles.

The present findings show that CSTs perform safely when given the opportunity. The higher preoperative CRP and age in consultant-led cases suggest selective allocation of complex or high-risk cases, which is appropriate. However, a large proportion of uncomplicated appendicectomies could be delegated to core trainees under supervision. Operative duration, complication rates, and readmissions did not differ significantly between operator grades. This supports supervised autonomy for core trainees, particularly when competency is demonstrated.

This study has several limitations that should be acknowledged. Firstly, it is a retrospective, observational study, which inherently carries the risk of selection bias and limits the ability to establish causality. Data was dependent on the accuracy and completeness of existing medical records, which may result in underreporting of complications or other relevant variables. Secondly, the sample size is relatively small, and therefore, the findings may not be fully generalizable to larger populations. Finally, the study is single-centred, reflecting the practices, training structure, and service pressures of one hospital. These factors may differ across institutions, limiting the broader applicability of the results.

Several novel strategies have been suggested to improve training opportunities for junior trainees. Laparoscopic appendicectomies have been shown to be a great model for trainees to act as trainers [20]. CSTs could greatly benefit from increased opportunities supported by registrars as trainers using appropriate cases. The use of virtual reality in laparoscopic training has shown good promise [21] and can be incorporated by trusts and deaneries as part of regular skills training. Low-cost simulation models incorporating artificial intelligence have been developed and trialled with great initial success [22]. Structured supervision, equitable case allocation, and emphasis on training are necessary to ensure CST fulfils its purpose of preparing trainees for higher surgical training.

## Conclusions

Registrars currently undertake the majority of appendicectomies in this district general hospital, while CSTs are under-represented despite performing comparably when involved. Our findings show that appendicectomies by CSTs are safe and effective. Future work should explore national trends and evaluate interventions to optimise operative exposure for core trainees.
